# Two novel bioassays useful for the quick assessment of chemical effects on the behavior of mosquito larvae (Culicidae) and adult earthworms (Lumbricidae)

**DOI:** 10.1016/j.mex.2022.101661

**Published:** 2022-03-07

**Authors:** Sanele Michelle Mnkandla, Patricks Voua Otomo

**Affiliations:** a Ecotoxicology Research Group, Department of Zoology and Entomology, Faculty of Natural and Agricultural Sciences, University of the Free State, Private Bag x13, Phuthaditjhaba, 9866, South Africa; b Ecotoxicology Research Group, Department of Applied Biology and Biochemistry, National University of Science and Technology, Bulawayo, Zimbabwe

**Keywords:** Behavioral changes, Chemical exposure, Mosquito larvae behavior, Earthworm escape distance

## Abstract

The behavior, i.e., observable response of an organism can serve as a useful indicator of environmental quality. In the absence of death and other sublethal effects, behavioral changes can represent early warning signs of toxic effects. In the present contribution, we present two novel bioassays useful for the quick assessment of chemical effects on the behavior of mosquito larvae (Culicidae) and adult earthworms (Lumbricidae). These bioassays could have applications in ecological risk assessment and laboratory based ecotoxicological testing. The novel assays are:

• A bioassay for the assessment of chemical toxicity on the swimming, breathing and resting behavior of Culicidae larvae.
• A bioassay for the assessment of chemical toxicity on the escape behavior of Lumbricidae in water.
• These bioassays were carried out using the systemic insecticide imidacloprid and documented the relatively rapid onset of the neurotoxic effects of imidacloprid to experimental organisms.

## Graphical abstract

**Figure:**
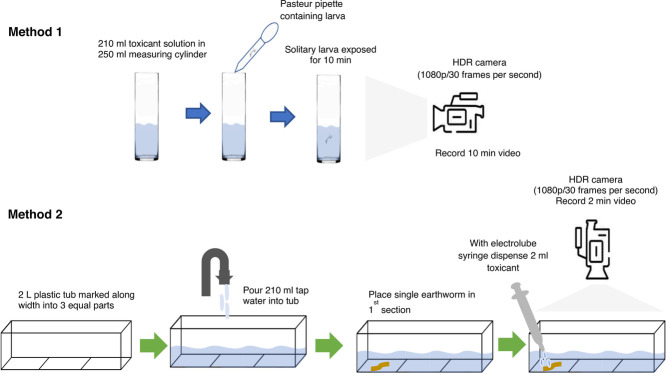

Specifications table

**Table:** 

Subject Area;	Agricultural and Biological Sciences
More specific subject area;	*Behavioral Ecotoxicology*
Method name;	• *Bioassay for the assessment of chemical toxicity on the swimming, breathing and resting behavior of Culicidae larvae* • *Bioassay for the assessment of chemical toxicity on the escape behavior of Lumbricidae in water*
Name and reference of original method;	Both methods are novel.
Resource availability;	*NA*

## Materials

*Bioassay for the assessment of chemical toxicity on the swimming, breathing and resting behavior of Culicidae larvae*

• Aphicide Plus® insecticide - containing 20g/L of Imidacloprid (IMI or N-{1-[(6-Chloro-3-pyridyl)methyl]-4,5-dihydroimidazol-2-yl}nitramide)
• Fourth, instars culicids larvae
• Distilled water
• 250 ml glass measuring cylinders (x 3)
• 5 ml plastic Pasteur pipettes (x 3)
• HD camera (1080p/30 frames per second)

*Bioassay for the assessment of chemical toxicity on the escape behavior of Lumbricidae in water*

• OECD artificial soil [10% sphagnum peat, 20% kaolin clay and 70% sand] [6]
• Cow dung (5 g per 10 worms per week)
• *Eisenia fetida* earthworms
• Paper towels
• Non-toxic food dye (Moir's Sky Blue)
• Permanent marker
• Distilled water
• 2-Liter rectangular plastic tubs [Length 220 mm, Width 162 mm, Height 76 mm] (x 4)
• 10 ml electrolube plastic syringes (x 4)
• HD camera (1080p/30 frames per second)
• Image J software (https://imagej.net/)

## Procedure

### Test organisms

#### Mosquito larvae

1. Collect fourth, instars culicids larvae from standing waters, in a relatively pristine environment with no history of chemical exposure.
2. Use in behavioral assessments within 24 hours.

#### Earthworms

1. Culture a population of *Eisenia fetida* earthworms on OECD artificial soil and feed with cow dung. Such a culture can also be purchased.
2. Prior to exposure, carefully hand-sort earthworms. Select adults, wash them in tap water and gently dab them with a paper towel to remove any dirt and excess water.

Assessment of chemical toxicity on the swimming, breathing and resting behavior of Culicidae larvae

1. Dilute Aphicide Plus in distilled water to make solutions of 1 and 2 mg IMI/L. Control solution (0 mg IMI/L), is distilled water. Depending on experimental design, more concentrations can be used, as long as they remain sublethal.
2. In separate measuring cylinders, fill each solution prepared in step 1 to the 210 ml mark.
3. Using a Pasteur pipette, place a single organism in each cylinder (at the surface of the water column).
4. Allow the solitary larva to acclimatize to each solution for 5 min, at room temperature, before filming (this step is optional because exposure starts the moment the organism enters the water).
5. Using the HD camera, film/record the larva in the water column for 10 min.
6. Repeat steps 3 – 5 twenty times at least, with a new larva each time.
7. Assess the following behavioral responses: breathing, swimming, and resting. Breathing occurs when the larvae are positioned upside-down with their breathing tubes (tail ends) protruding beyond the surface of the water column; Swimming is defined as the free exploration of the water column by the larvae; and resting is the behavior typified by the larvae lying motionless at the bottom of the water column.

Assessment of chemical toxicity on the escape behavior of Lumbricidae in water

1. Dilute Aphicide Plus in distilled water dyed with blue food dye (1:12 v/v), and prepare solutions of 5, 10 and 20 mg IMI/L. Control solution (0 mg IMI/L) is made of the blue food dye and distilled water. The coloration of the treatments is necessary to visualize pesticide movement in the test container.
2. Using the permanent marker, mark each rectangular plastic tub along the width, to divide the base of the container into three equal parts. Marking helps in visualizing the movement of earthworms in the tubs during experimentation. This step is optional.
3. Fill each tub with 210 ml of tap water.
4. Place a single earthworm in the first section of the tub.
5. Using the electrolube plastic syringe, pump 2 ml of each solution (separately) directly onto the earthworm.
6. Using the HD camera, record the worm's response on video for 2 min from a high vantage point.
7. Replicate steps 3-6 at least ten times.
8. To measure the initial escape distance covered by the earthworm, i.e., the distance travelled by the worm before stopping, backtracking, or recoiling, after the release of the imidacloprid solution:
i) pause the video on the last frame showing this maximum distance travelled.
ii) take a screenshot of that frame.
iii) using the Image J software, upload the screenshot and measure the distance (in pixels, which is then converted to millimeters using the actual size of the plastic tub as a reference).
iv) Measure the distance traveled by the tip of the head (prostomium) from the beginning to the end of the initial flight response.
9. To calculate escape speed, divide the distance travelled by the earthworm by the time it took to travel that distance [i.e., escape speed = distance travelled (mm)/ duration of travel (s)]. Duration of travel is calculated using the video time stamp.

## Data analysis

1. In the present case, behavioral data of the Culicidae (i.e., time spent swimming, resting, or breathing) were non-parametric and consequently analyzed using the Kruskal-Wallis ANOVA followed by Dunn's comparison test.
2. One Way-ANOVA followed by Turkey post-hoc test was used to analyze the distance and speed data of the earthworms.
3. All data were analyzed using GraphPad Prism (GraphPad Prism version 5.00 for Windows, GraphPad Software, San Diego, CA, USA, www.graphpad.com). The level of significance was p < 0,05.

## Method validation

### Assessment of chemical toxicity on the swimming, breathing and resting behavior of Culicidae larvae

We investigated the effect of imidacloprid exposure on the breathing, swimming, and resting behavior of Culicidae larvae. Briefly, Aphicide Plus pesticide (containing Imidacloprid) was diluted in distilled water and solutions of 1 and 2 mg IMI/L were made (control, 0 mg IMI/L, distilled water only). The solutions were separately filled into measuring cylinders, prior to placing solitary larvae in the cylinders. The larvae were then filmed using an HD camera for 10 min.

### Breathing

**Fig. 1:**
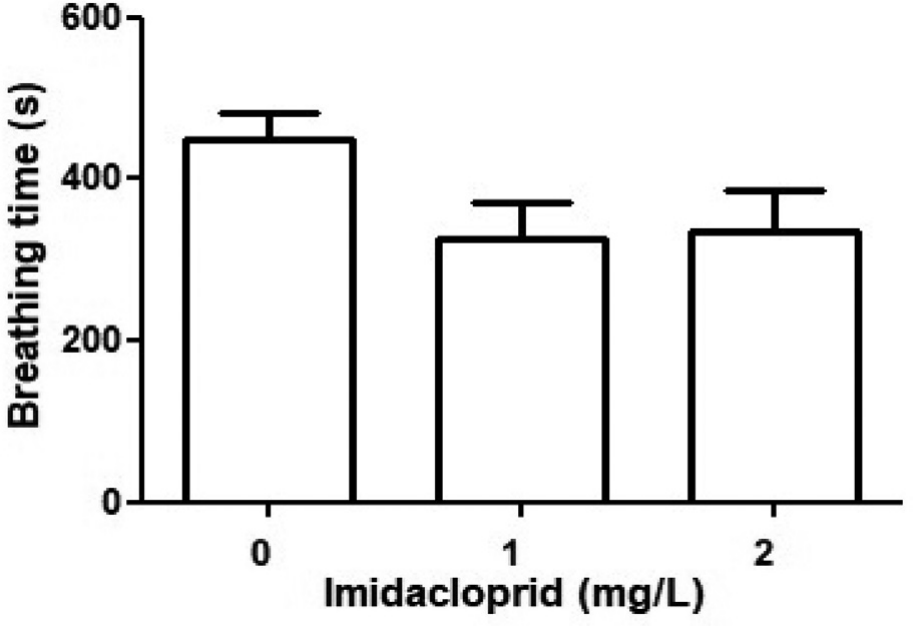
The effects of imidacloprid on the breathing rate of culicids. Solitary culicids were exposed to 0, 1 and/or 2 mg IMI/L and recorded by HD camera. The total time recorded = 600 seconds per culicid larvae and per concentration. All values are means ± SD, n = 20 culicid larvae per treatment.

Fig. 1 shows the effects of imidacloprid on the breathing behavior of the Culicidae larvae. In the 1 mg IMI/L and 2 mg IMI/L imidacloprid treatments, the culicids spent 52% and 56% (respectively) of their time breathing, which was much lower than the culicids in the control (0 mg IMI/L), which spent 75% of their time breathing. These data indicate a slight decrease in breathing after the addition of imidacloprid, albeit not significantly different within the concentration range assessed (p > 0,05).

### Swimming

**Fig. 2:**
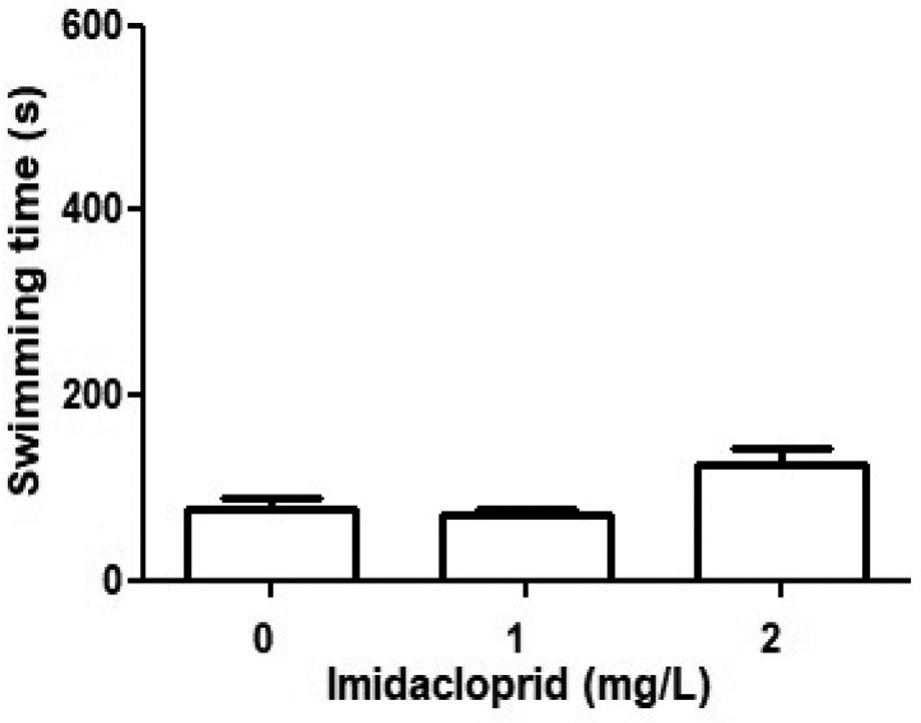
The effects of imidacloprid on the swimming rate of culicids. Solitary culicids were exposed to 0, 1 and/or 2 mg IMI/L and recorded by HD camera. The total time recorded = 600 seconds per culicid larvae and per concentration. All values are means ± SD, n = 20 culicid larvae per treatment.

Swimming was the least common behavior displayed by the culicids, amounting to 13% of the time in the control, and 11% and 21% of the time in the 1 mg IMI/L and 2 mg IMI/L imidacloprid treatments, respectively (Fig. 2). Despite there being no statistical difference in the effect of imidacloprid on the swimming behavior of culicids within the concentration range assessed (p > 0,05), the near doubling of the swimming time between 1mg IMI/L (11%) and 2 mg IMI/L (21%) suggests that with an increasing presence of imidacloprid in the testing medium, the culicid larvae spend more time swimming (perhaps floating away or transitioning to the bottom of the water column as the next finding would imply).

### Resting

**Fig. 3:**
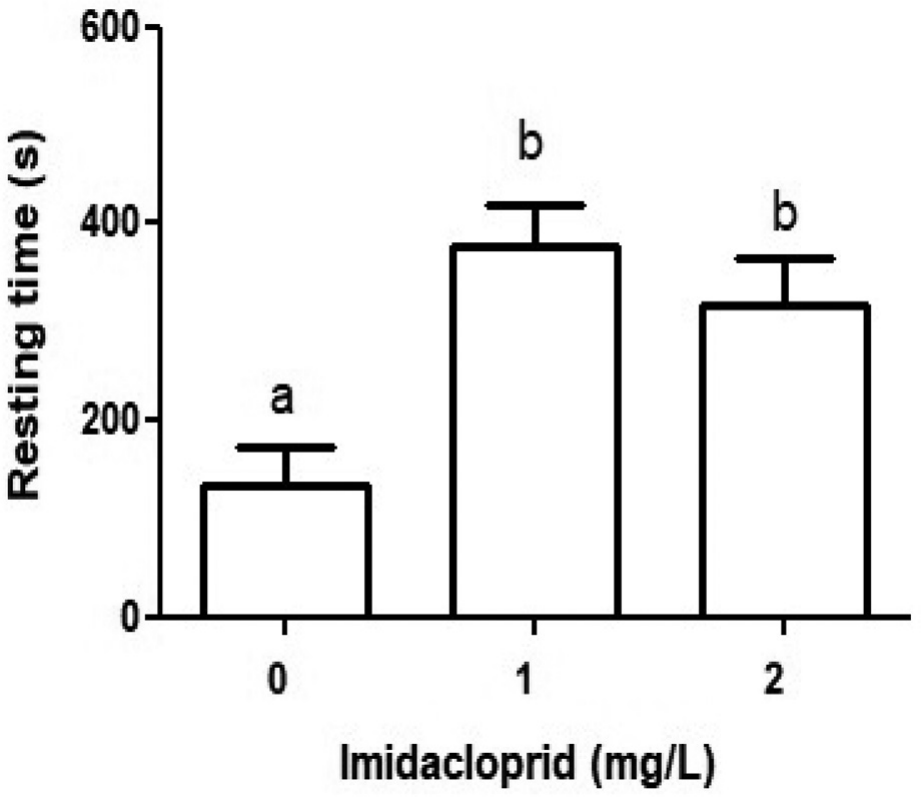
The effects of imidacloprid on the resting rate of culicids. Solitary culicids were exposed to 0, 1 and/or 2 mg IMI/L and recorded by HD camera. The total time recorded = 600 seconds per culicid larvae and per concentration. All values are means ± SD, n = 20 culicid larvae per treatment. Letters indicate significant difference between groups (p<0,05).

Fig. 3 shows that the resting behavior was significantly higher in both imidacloprid treatments relative to the control (p < 0,05). This suggests that imidacloprid caused the culicid larvae to spend considerably less time at both the surface and within the water column (breathing and swimming) and significantly more time resting motionless at the bottom. This could be explained based on the mode of action of this pesticide on the nervous system. Imidacloprid, like other neonicotinoids, binds irreversibly to postsynaptic nicotinic acetylcholine receptors (nAChRs) as it cannot be degraded by acetylcholinesterase [8,13]. The organisms’ inability to break down this xenobiotic neurotransmitter ultimately leads to over-stimulation of the central nervous system, causing uncontrolled muscular contraction, paralysis, and death- if the effects are sustained [8,13].

### Assessment of chemical toxicity on the escape behavior of Lumbricidae in water

*Eisenia fetida* earthworms were exposed to imidacloprid stock solutions of 0 (control), 5, 10 and 20 mg IMI/L, dyed using a non-toxic food dye for visualization of pesticide movement. In a plastic tub marked into three equal parts, and filled 210 ml of tap water, a single worm placed in the first section of the tub. Each pesticide solution was then directly pumped directly onto the worm and the worm's response was recorded using an HD camera, for 2 min.

### Initial escape distance

**Fig. 4:**
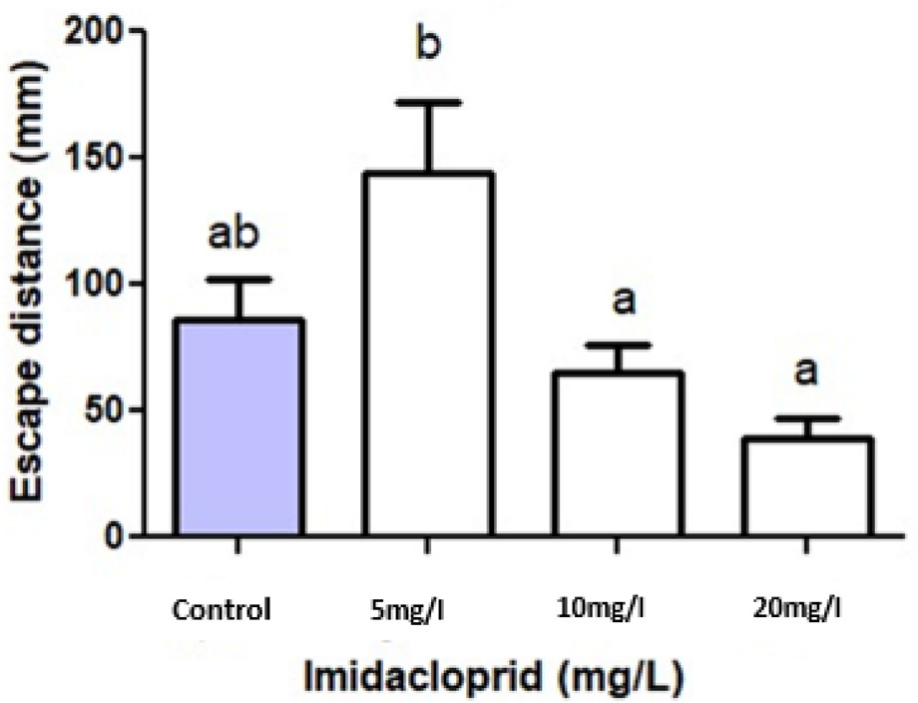
Initial escape distance of the earthworm *Eisenia fetida* (in mm) after topical exposure to different imidacloprid concentrations via 10 ml electrolube plastic syringes. All values are means ± SD, n = 10 earthworms per treatment. Different letters indicate statistical differences (p < 0,05).

Figure 4 shows that after topical application of imidacloprid to the earthworms, the initial escape distance, from the starting point to the point the earthworms stopped and recoiled, was significantly longer after exposure to the 5 mg/L solution when compared to the control, 10 and 20 mg/L concentrations (p < 0,05). The distance covered in reaction to the 5 ml/L solution was, however, comparable to that found after topical application of the control (p > 0,05). Escape distance following exposure to the 10 and 20 mg/L solutions did not differ significantly from the control (p > 0,05). Nevertheless, overall, as the imidacloprid concentration increased, the earthworm initial escape distance decreased. These findings suggest that initially, in the lower concentration of imidacloprid, the earthworms could sense it and act by displaying a flight behavior. However, as the concentration of imidacloprid increased, the worms seemed to show less of that behavior, only escaping to shorter distances. In the two highest concentrations of imidacloprid, the initial escape distance was even shorter than observed in the control, albeit insignificantly. It is possible that the higher concentrations of imidacloprid exerted more significant adverse effects on the central nervous system and locomotion of the worms than the lowest one, thus preventing the worms from escaping further [8,13].

### Initial escape speed

**Fig. 5:**
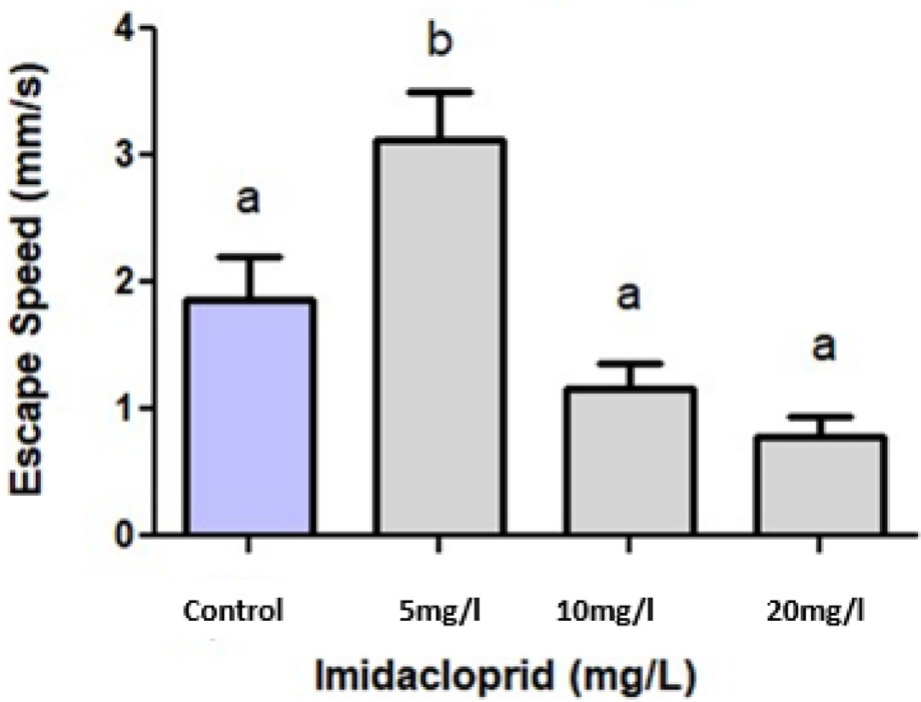
Initial escape speed of the earthworm *Eisenia fetida* (in mm/s) after topical exposure to different imidacloprid concentrations via 10 ml electrolube plastic syringes. All values are means ± SD, n = 10 earthworms per treatment. Different letters indicate statistical differences (p < 0,05).

The speed of initial flight by *E fetida* is shown in Figure 5. The results showed a similar trend with those of escape distance (Figure 5). Following topical exposure of the earthworms to imidacloprid, the speed of escape, from the starting point to the point the earthworms stopped and recoiled, was significantly higher only after exposure to the 5 mg/L solution (p < 0,05), relative to the other concentrations. Overall, higher imidacloprid concentrations caused a decrease in the earthworm initial reaction speed. Imidacloprid has been found to negatively affects flight behavior, locomotion and decision making in several other invertebrates such as beetles [10,^14^,7], honeybees [12,^5^,^11^,15], harlequin flies [1] and vertebrates such as amphibians [4,9] and zebrafish [2]. A standardized earthworm avoidance protocol already exists for the soil environmental [3]. The protocol in question recommends a test duration of 2 days to establish the avoidance response of the test subjects. In the approach presented here, we establish such a response in less than 2 minutes. This novel protocol is therefore substantially quicker to perform and less laborious.

## Declaration of competing interest

The authors declare that they have no known competing financial interests or personal relationships that could have appeared to influence the work reported in this paper.

## References

[1] Azevedo-Pereira H., Lemos M., Soares A. (2011). Effects of imidacloprid exposure on *Chironomusriparius Meigen* larvae: linking acetylcholinesterase activity behavior. Ecotoxicol Environ Saf.

[2] Crosby E.B., Bailey J.M., Oliveri A.N., Levin E.D. (2015). Neurobehavioral impairments caused by developmental imidacloprid exposure in zebrafish. Neurotoxicology.

[3] ISO, 2008. International organization for standardization 17512-1 - Soil quality - Avoidance test for determining the quality of soils and effects of chemicals on behaviour - Part 1: Test with earthworms (*Eisenia foetida* and *Eisenia andrei*).

[4] Lee-Jenkins S.S.Y., Robinson S.A. (2018). Effects of neonicotinoids on putative escape behavior of juvenile wood frogs (*Lithobates sylvaticus*) chronically exposed as tadpoles. Environ. Toxicol. Chem..

[5] Medrzycki P., Montanari R., Bortolotti I., Sabatini A.G., Maini S., Porrini C. (2003). Effects of imidacloprid administered in sub-lethal doses on honey bees' behaviour. Bull. Insectology..

[6] OECD (2004).

[7] Papachristos D.P., Milonas P.G. (2008). Adverse effects of soil applied insecticides on the predatory coccinellid *Hippodamia undecimnotata* (Coleoptera: Coccinellidae). Biol. Control..

[8] SERA, 2005. Imidacloprid – Human Health and Ecological Risk Assessment – Final Report; Prepared for USDA, Forest Service, USA (SERA TR 05-43-24-03a).

[9] Sievers M., Hale R., Swearer S.E., Kirsten P.M. (2018). Contamination mixture interect to impair predator-avoidance behaviour and survival in a larval amphibian. Ecotoxicol. Environ. Saf..

[10] Smith S.F., Krischik V.A. (1999). Effects of systematic Imidacloprid on Coleomegilla maculate (Coleoptera: Coccinellidae). Environ. Entomol..

[11] Suchail S., Guez D., Belzunces L.P. (2001). Discrepancy between acute and chronic toxicity induced by imidacloprid and its metabolites in *Apis mellifera*. Environ. Toxicol. Chem..

[12] Thorne B.L., Breish L.N. (2001). Effects of sublethal exposure to imidacloprid on subsequent behaviour of subterranean termites. *Reticulitermes virginicus*. J. Entomol..

[13] Tomizawa M., Casida J.E. (2005). Neonicotinoid insecticide toxicology: mechanisms of selective action. Annu. Rev. Pharmacol. Toxicol.

[14] Vincent C.H., Ferran A.N., Guige L.U., Gambier J.A., Brun J.A. (2000). Effects of Imidacloprid on Harmonia axyridis (Coleoptera: Coccinellidae) larval biology and locomotory behavior. Eur. J. Entomol..

[15] Zhang Z.Y, Li Z., Huang Q., Yan W.Y., Zhang L.Z., Zeng L.J. (2020). Honeybees (*Apis mellifera*) modulate dance communication in response to pollution by imidacloprid. J. Asia. Pac. Entomol..

